# Pharmacogenetic Modulation of Orexin Neurons Alters Sleep/Wakefulness States in Mice

**DOI:** 10.1371/journal.pone.0020360

**Published:** 2011-05-27

**Authors:** Koh Sasaki, Mika Suzuki, Michihiro Mieda, Natsuko Tsujino, Bryan Roth, Takeshi Sakurai

**Affiliations:** 1 Department of Molecular Neuroscience and Integrative Physiology, Faculty of Medicine, Kanazawa University, Kanazawa, Japan; 2 Pharmacology and Medicinal Chemistry, UNC Chapel Hill Medical School, Chapel Hill, North Carolina, United States of America; Institut National de la Santé et de la Recherche Médicale, France

## Abstract

Hypothalamic neurons expressing neuropeptide orexins are critically involved in the control of sleep and wakefulness. Although the activity of orexin neurons is thought to be influenced by various neuronal input as well as humoral factors, the direct consequences of changes in the activity of these neurons in an intact animal are largely unknown. We therefore examined the effects of orexin neuron-specific pharmacogenetic modulation *in vivo* by a new method called the *D*esigner *R*eceptors *E*xclusively *A*ctivated by *D*esigner *D*rugs approach (DREADD). Using this system, we successfully activated and suppressed orexin neurons as measured by Fos staining. EEG and EMG recordings suggested that excitation of orexin neurons significantly increased the amount of time spent in wakefulness and decreased both non-rapid eye movement (NREM) and rapid eye movement (REM) sleep times. Inhibition of orexin neurons decreased wakefulness time and increased NREM sleep time. These findings clearly show that changes in the activity of orexin neurons can alter the behavioral state of animals and also validate this novel approach for manipulating neuronal activity in awake, freely-moving animals.

## Introduction

The neuropeptides orexin A and orexin B, also called hypocretin 1 and hypocretin 2, are produced in neurons located in the lateral hypothalamic area (LHA). Disruption of orexin signaling has been shown to result in narcolepsy in mice, dogs, and humans, highlighting the critical roles of orexins in the maintenance of wakefulness and in the regulation of rapid eye movement (REM) sleep.

Intracerebroventricular administration of orexin into animals has been shown to increase the amount of wakefulness, accompanied by decreases in the amount of both NREM and REM sleep [1]. These effects are likely mediated by activation of monoaminergic neurons in the brain stem, which express high numbers of orexin receptors [2]. Although orexins play a key role in the sleep/wakefulness control system, with the activity of orexin neurons increasing during wakefulness and decreasing during sleep [3], [4], [5], it is as yet unclear how awake, freely-moving animals will react to changes in the activity of orexin neurons.

Since orexin neurons act as stabilizers of sleep/wakefulness states, receiving input from various brain regions [2], changes in their activity of these neurons in each vigilance state may occur secondarily to changes in sleep/wakefulness states. Alternatively, the activity of orexin neurons may actively affect the vigilance states of animals. The latter proposal is supported by findings showing that the discharge of orexin neurons increases before the end of both NREM and REM sleep, thereby preceding by several seconds a return to wakefulness [4]. Orexin neurons may therefore act as integrators, receiving input from other brain regions and, accordingly, actively regulating vigilance states [2].

This hypothesis is supported by the finding that optogenetic excitation of orexin neurons results in increases in the probability of an awakening event during both NREM and REM sleep, although this excitation did not change the total amount of wakefulness [6], [7]. However, the consequences of the acute inhibition of orexin neurons in freely behaving mice remain unknown.

In this study, we utilized a pharmacogenetic technique called “*D*esigner *R*eceptors *E*xclusively *A*ctivated by *D*esigner *D*rugs (DREADD)” [8]. This method utilizes extrinsic muscarinic receptors, (hM3D_q_ for excitation and hM4D_i_ for inhibition), that have lost their affinity for endogenous acetylcholine but can still be activated by a synthetic and pharmacologically inert ligand, clozapine-N-oxide (CNO). Activation of G_q_-coupled hM3D_q_ by CNO was previously shown to activate neurons through phospholipase C-dependent (PLC) mechanisms [9]_ENREF_9. We confirmed the effect of hM3D_q_ in orexin neurons by calcium imaging (Fig. S1A)_ENREF_9. CNO can also stimulate G_i/o_-coupled hM4D_i_ receptors, thereby activating inwardly rectifying potassium 3 (Kir3) channels, resulting in membrane hyperpolarization and neuronal silencing [8], [10]. We also confirmed the inhibitory effect of hM4D_i_ in orexin neurons by calcium imaging [11] (Fig. S1B).

**Figure 1:**
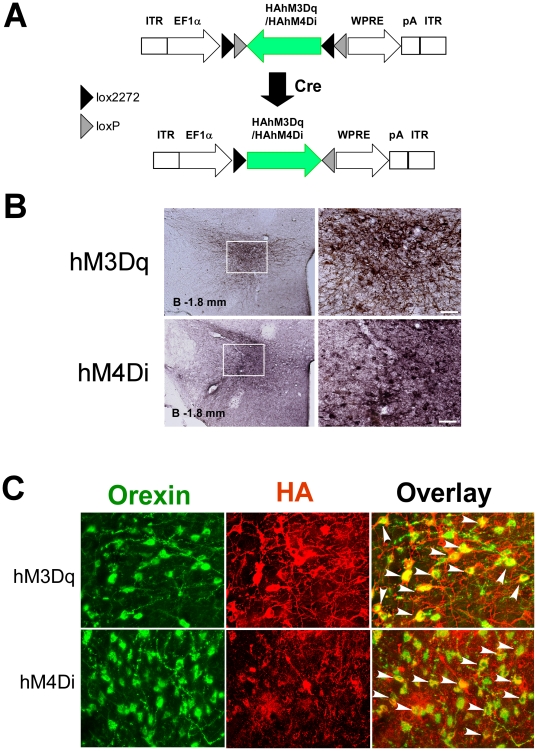
Specific expression of hM3D_q_ and hM4D_i_ in orexin neurons in mice. A, Schematic representation of double-floxed Cre-dependent AAV vector expressing hM3D_q_ and hM4D_i_ under control of EF-1α promoter (rAAV-DIO-HAhM3Dq and rAAV-DIO-HAhM4Di). DIO, double-floxed inverted open reading frame; ITR, inverted terminal repeat; WPRE, woodchuck hepatitis virus post-transcriptional regulatory element. B, Coronal brain sections at the level of LHA (bregma-1.8 mm), prepared from *orexin-cre* mice expressing hM3D_q_ or hM4D_i_ following injection of Cre-dependent rAAV-DIO-HAhM3Dq or rAAV-DIO-HAhM4Di. Sections were stained with anti-HA antibody. Scale bars, 40 µm. C, Upper panels, Coronal section of LHA prepared from *orexin-cre* mice expressing hM3D_q_ and double stained with anti-orexin antibody (green) and anti-HA antibody (red), showing that most of orexin-ir neurons express HAhM3D_q_. Lower panels, Coronal section of LHA prepared from *orexin-cre* mice expressing hM4D_i_ and double stained with anti-orexin antibody (green) and anti-HA antibody (red), showing that most of orexin-ir neurons express HAhM4D_i_.

Using transgenic mice in which Cre-recombinase is expressed exclusively in orexin neurons (*orexin-cre* transgenic mice) [12], these receptors were expressed solely on orexin neurons by transfection with adeno-associated virus (AAV) vectors utilizing the flip-excision method, which restores the open reading frame in a Cre-dependent manner [13], [14] (Fig. 1A). As stimulation of GPCRs with a specific ligand has a longer effect on cellular signaling than optical stimulation of chanelrhodopsin 2 (ChR2) ion channels, the DREADD system can facilitate the examination of the chronic effects of modulating the activity of orexin neurons. In addition, since CNO is a stable substance that can cross the blood brain barrier, it can be administered intraperitoneally with minimum interference with the behavioral state in freely-moving animals. Our results demonstrated that activation of orexinergic tone results in increased wakefulness time, while inhibition of these neurons results in reduced wakefulness.

## Results

### Expression of hM3D_q_ and hM4D_i_ in Orexin Neurons

To obtain selective pharmacogenetic control of orexin neurons *in vivo*, we injected a recombinant adeno-associated virus (AAV2) containing a doubly-floxed inverted open reading frame encoding hM3D_q_ (rAAV-DIO-HAhM3Dq) or hM4D_i_ (rAAV-DIO-HAhM4Di) (Fig. 1A) into the LHA of *orexin-cre* transgenic mice, in which Cre-recombinase is highly and specifically expressed in orexin-producing neurons. These AAV vectors facilitate the expression of N-terminally HA-tagged hM3D_q_ or hM4D_i_ exclusively in Cre-expressing cells (see Methods).

Immunohistochemical staining of brain slice preparations showed that HA- immunoreactivity was observed exclusively in the LHA region of *orexin-cre* transgenic mice injected with rAAV-DIO-HAhM3Dq or rAAV-DIO-HAhM4Di (Fig. 1B). HA- immunoreactivity was robustly observed in dendrites and axons as well as cell bodies of the LHA neurons. No detectable staining was observed in wild type mice infected with the same viruses (not shown). These observations suggest that hM3D_q_ and hM4D_i_ were specifically expressed in the LHA in a Cre-dependent manner.

Moreover, double staining with anti-orexin and anti-HA antibodies revealed HA-immunoreactivity in most orexin-producing neurons throughout the hypothalamus in *orexin-cre* mice bilaterally injected with rAAV-DIO-HAhM3Dq or rAAV-DIO-HAhM4Di (Fig. 1C), confirming that hM3D_q_ and hM4D_i_ were expressed exclusively in orexin-producing neurons. We examined the expression rates of hM3D_q_ and hM4D_i_ throughout the hypothalamus and found that 78.2±8.1% and 73.2±10.9% of orexin-immunoreactive neurons were positive for HA staining, when rAAV-DIO-HAhM3Dq or rAAV-DIO-HAhM4Di was injected into *orexin-cre* mice, respectively (n = 3 each). We did not detect any ectopic expression of hM3Da and hM4Di in non-orexin-producing neurons.

### Modulation of Orexinergic Activity by Intraperitoneal CNO Administration

We implanted thin silicone tubes into the peritoneal space of mice so that we could administer CNO with minimal perturbation. The effects of excitatory hM3D_q_ were examined during the light period, at the time of day when orexinergic activity is minimal (Fig. S2, Experiment 1). Following intraperitoneal injection of CNO at 13:00 [zeitgeber time (ZT) 4], into *orexin-cre* mice expressing hM3D_q_, the animals were sacrificed and fixed at 15:00 (ZT6). Similarly, the effect of CNO in mice expressing hM4D_i_ was examined during the dark period, when orexinergic activity is maximal (Fig. S2, Experiment 2). CNO was injected intraperitoneally, just before the light was turned off at 21:00 (ZT12), into *orexin-cre* mice expressing hM4D_i_, and the mice were sacrificed and fixed at 23:00 (ZT14) for immunostaining. We used wild type mice injected with rAAV-DIO-HAhM4Di as controls. Hypothalamic slices of these mice were examined by double staining with anti-orexin and anti-Fos antibodies to assess the activity of these orexin neurons.

**Figure 2:**
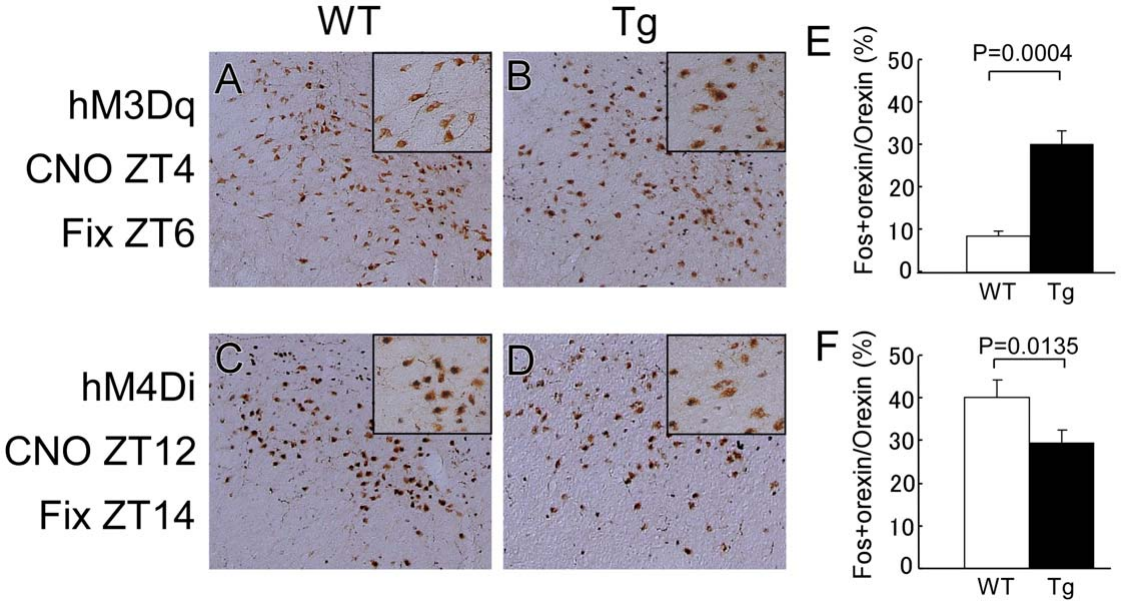
Activation or inhibition of orexin neurons by DREADD. A–D, Representative images of Fos expression in orexin neurons, as shown by double staining of the LHA regions of wild type (A, C) and *orexin-cre* mice (B, D) injected with cre-activatable AAV carrying hM3D_q_ (A, B) or hM4Di (C, D) (rAAV-DIO-HAhM3Dq or rAAV-DIO-HAhM4Di) after injection of CNO. A, Only a small numbers of Fos-IR nuclei were observed in orexin-immunoreactive neurons from wild type mice administered CNO at 13:00 (ZT4) and killed at 15:00 (ZT6). Inset, high power view. B, Number of Fos- and orexin-double-positive neurons was higher in *orexin-cre* than in wild type (A) administered CNO at ZT4 and killed at ZT6. Inset, high power view. C, Many Fos- and orexin-double positive neurons were observed in wild type mice administered CNO at ZT12 and killed at ZT14. Inset, high power view. D, Fewer Fos- and orexin-double-positive neurons were observed in *orexin-cre* than in wild type mice administered CNO at ZT12 and killed at ZT14. Inset, high power view. E, Numbers of double-positive neurons at ZT6 in wild type and *orexin-cre* transgenic mice injected with hM3D_q_ virus and administered CNO (N = 5). F, Numbers of double-positive neurons at ZT14 in wild type and *orexin-cre* transgenic mice injected with hM4D_i_ virus and administered CNO (N = 5).

In wild type mice, in which orexin neurons did not express hM3D_q_ or hM4D_i_, the number of double-labeled neurons (orexin-positive neurons with Fos-positive nuclei) showed marked diurnal fluctuation, with a lower level of double-labeling at ZT6 (8.4±1.1%) and higher level at ZT14 (44.7±4.3%) (Fig. 2A, C). These findings are consistent with the results observed in unmanipulated wild type mice (not shown) and previously reported in rats [15].

When we examined rAAV-DIO-HAhM3Dq-injected *orexin-cre* mice, we observed a 261.1% increase in double-labeled neurons when compared with wild type controls in the light periods (30.4±2.86% vs 8.4±1.1%) (Fig. 2A, B, E). Similarly, when we assayed rAAV-DIO-HAhM4Di-injected *orexin-cre* mice, we observed a 34.0% decrease in double-labeled neurons compared with wild type mice in the dark periods (29.5±3.1% vs 44.7±4.3%) (Fig. 2C, D, F). These observations demonstrate that the DREADD system used in this study appropriately modulates the activity of orexin neurons.

### Modulation of Orexinergic Activity Alters Behavioral States in Mice

To examine the effect of hM3D_q_ stimulation of orexin neurons on sleep/wakefulness states, we first administered CNO or saline intraperitoneally to *orexin-cre* mice, in which orexin neurons specifically express hM3D_q_, at ZT4 (Fig. S2, Experiment 1). The sleep/wakefulness states of these mice were monitored by simultaneous EEG/EMG recordings. As a control, we treated the same mice with saline on separate experimental days.

**Figure 3:**
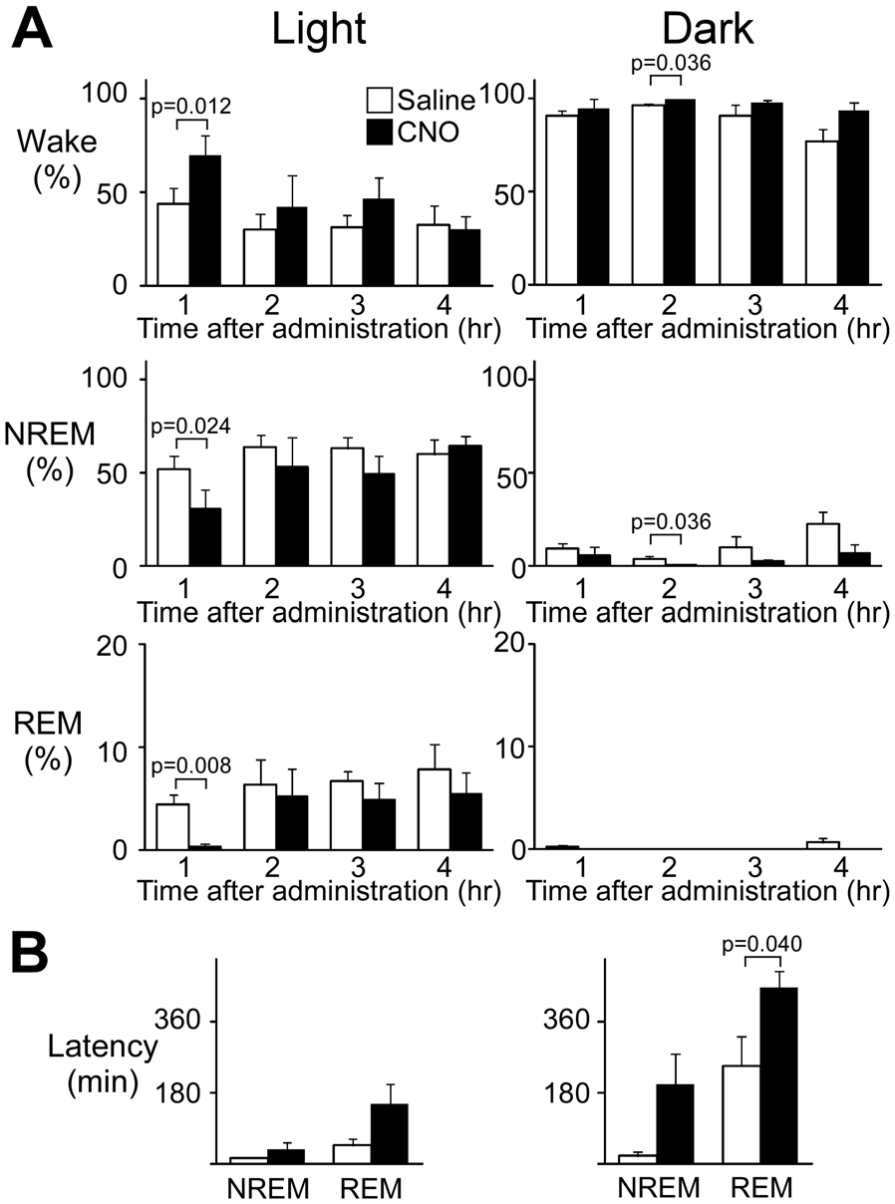
Effect of stimulation of orexinergic tone by hM3D_q_ on vigilance states of mice during light and dark periods (left and right panels, respectively). A. Hourly analysis of sleep/wake states in transgenic and wild-type mice, both injected with rAAV-DIO-HAhM3Dq, after administration of CNO at ZT4 or ZT12. Amounts of wakefulness (Wake, upper panels), NREM sleep (middle panels), and REM sleep (lower panels) are shown. B, Latency to NREM sleep and REM sleep after CNO administration (N = 6 for transgenic mice, N = 5 for wild-type mice).

We found that the percent of wakefulness during the one hour after CNO administration was significantly greater (69.5±12.4% vs 43.8±7.9%, t_5_ = -3.826, p = 0.012) and NREM time was significantly shorter (30.2±10.3% vs 51.9±7.0%, t_5_ = 3.182, p = 0.024) in CNO-treated conditions than in saline-injected control conditions (Fig. 3A, left panels, Fig. S3). These results suggest that stimulation of orexin neurons results in increased wakefulness time, accompanied by decreased NREM sleep time. We also observed a significant decrease in REM sleep time (0.31±0.31% vs 4.33±1.0%, t_5_ = 4.202, p = 0.008).

We also examined the effect of stimulation during the dark period to determine whether the pharmacogenetic activation of orexin neurons can enhance the effect of endogenous orexinergic activity (Experiment 2, Fig. S2). We administered CNO or saline intraperitoneally to *orexin-cre* mice, in which orexin neurons specifically express hM3D_q_, at ZT12 (Fig. S2, Experiment 2). We observed a modest effect on sleep/wakefulness states (Fig. 3, right panels). In a one-two hour time window following the injection, the wakefulness time was significantly longer in CNO-treated *orexin-cre* mice (99.9±0.13% vs 96.3±1.1%, t_5_ =  = -3.098, p = 0.036). Also, we observed that latency from wakefulness to REM sleep was longer in CNO-treated mice than in saline-treated controls (448.2±38.5 min vs 248.1±75.1 min, t_5_ = -3.003, p = 0.040; Fig 3B, right panel). These observations suggest that pharmacogenetic activation of orexin neurons affects sleep/wakefulness states in both the light and dark period.

**Figure 4:**
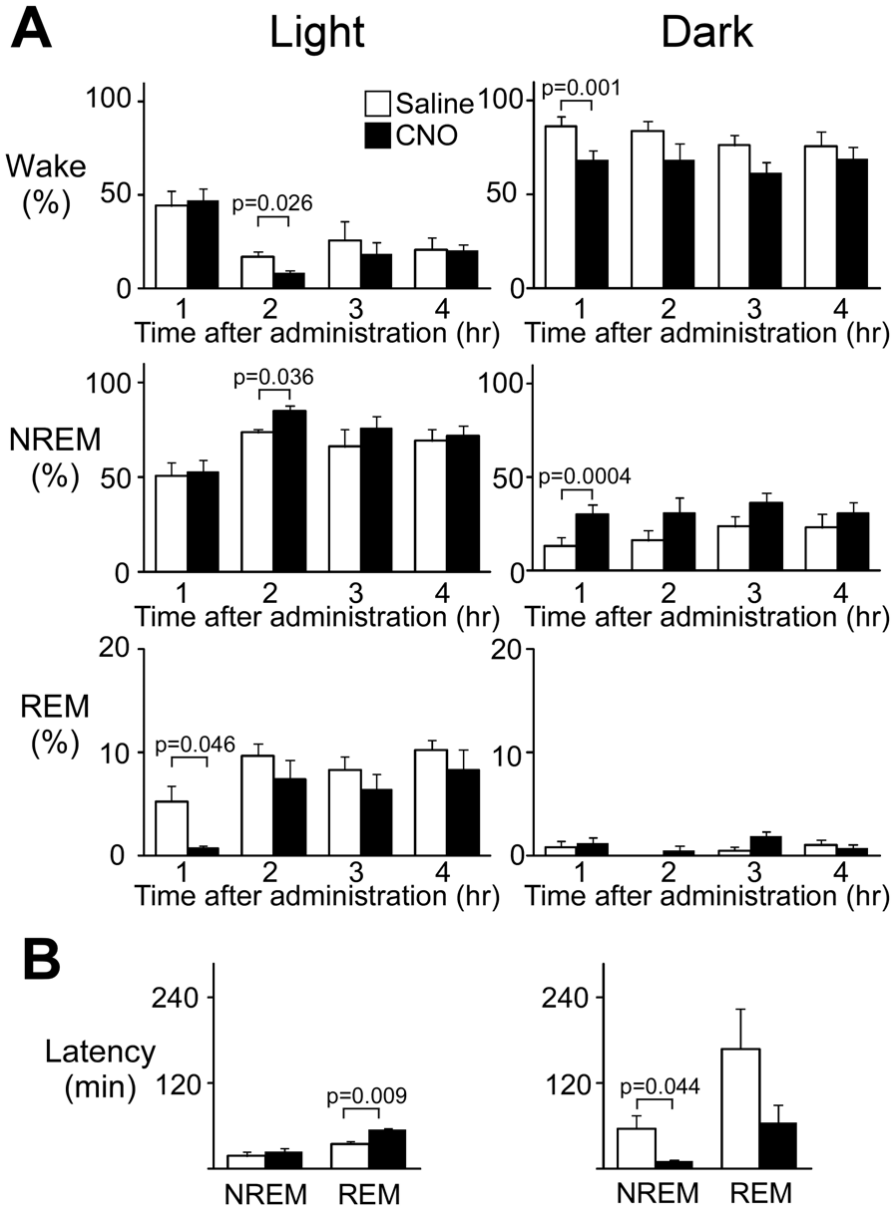
Effect of inhibition of orexinergic tone by hM4D_i_ on vigilance states of mice during light and dark periods (left and right panels, respectively). A. Hourly analysis of sleep/wake states in transgenic and wild-type mice, both injected with rAAV-DIO-HAhM4Di, after administration of CNO at ZT4 or ZT12. Amounts of wakefulness (Wake, upper panels), NREM sleep (middle panels), and REM sleep (lower panels) are shown. B. Latency to NREM sleep and REM sleep after CNO administration (N = 5).

Next, to test the effects of inhibition of orexin neurons on sleep/wakefulness states, we administered CNO or saline intraperitoneally to *orexin-cre* mice, in which orexin neurons specifically express inhibitory hM4D_i_, at ZT12 (Fig. S2, Experiment 2). The percent wakefulness during the one hour after CNO administration was significantly shorter (69.0±5.0% vs 86.6±5.0%, t_5_ =  = 6.492, p = 0.001), while NREM sleep time was significantly longer (29.9±5.1% vs 12.7±4.8%, t_5_ = -8.334, p = 0.0004), in CNO-treated mice than in controls (Fig. 4A, right pannels, Fig. S4). These observations suggest that inhibition of orexin neurons increases NREM time, accompanied by a decrease in wakefulness time. We observed a slight increase in REM sleep time, although this difference was not significant. The latency to NREM sleep was significantly shorter in CNO-treated mice as compared with controls (10.8±2.4 min vs 56.8±18.6 min, t_5_ = 2.689, p = 0.044; Fig. 4B, right pannel).

We also examined whether inhibition in the light period can still affect the sleep/wakefulness states of mice (Experiment 1, Fig. S2). We administered CNO or saline intraperitoneally to *orexin-cre* mice, in which orexin neurons specifically express hM4D_i_, at ZT4. We observed a modest effect on sleep/wakefulness states (Fig. 4, left panels). At one-two hours after injection, the wakefulness time was significantly shorter (8.1±1.4% vs 17.0±2.3%, t_5_ = 3.437, p = 0.026) and NREM sleep was longer in CNO-treated mice (84.6±3.0 min vs 73.4±1.5 min, t_5_ = −3.106, p = 0.036). Interestingly, REM sleep was significantly reduced in the first hour following CNO administration (0.67±0.3% vs 5.22±1.5%, t_5_ = 2.868, p = 0.046), whereas latency to REM was significantly increased in CNO-treated mice (55.5±1.9 min vs 34.6±4.8 min, t_5_ = −4.813, p = 0.009) (Fig. 4B, left panels).

## Discussion

The expression of Fos, a marker of neuronal activity, in orexin neurons in rats is increased during the dark, the period during which rats are active and the awake state is dominant [15]. Moreover, orexin level in CSF peaks during dark period and decreases during the light period, during which rats are inactive and the sleep state is dominant [16]. In addition, *in vivo* recording studies have shown changes in orexin neuronal activity across the sleep-wake cycle [3], [4], [5]. Taken together, these studies indicate that orexin neurons fire during active waking, have decreased their discharge rates during quiet waking, and virtually cease discharge during both REM and NREM sleep.

The main effector sites for orexin are thought to be the monoaminergic and cholinergic neurons in the brain stem. The discharge rates of these neurons are strongly associated with sleep and wakefulness, in that they fire tonically during awake periods, less during NREM sleep, and are almost completely quiescent during REM sleep [17], a pattern similar to the discharge of orexin neurons. Orexin neurons and monoaminergic neurons in the brain stem, including the locus coeruleus and dorsal raphe nucleus, have a reciprocal interaction constituting a feedback loop, thereby maintaining the activity of monoaminergic neurons and perhaps explaining the similar discharge patterns of orexin neurons and monoaminergic neurons [2]. Wake-active monoaminergic centers are also influenced by inhibitory projections from the preoptic area, especially the ventrolateral preoptic area (VLPO) [18]. Sleep may be maintained by VLPO sleep-active neurons, which send inhibitory projections to both the hypothalamic orexin neurons and brain stem monoaminergic neurons [19], [20]. These neural circuits may be important for the stability of wakefulness [2]. We therefore performed this study to determine the consequences of acutely changing orexinergic tone, which is thought to be important in modulating these circuits.

A prior study wherein optogenetic excitation of orexin neurons in mice was performed demonstrated an increased probability of transitions to wakefulness from NREM or REM sleep in mice [7]. The consequence(s) of the acute inhibition of orexin neurons in freely behaving mice remain unknown. Although halorhodopsin-mediated optogenetic inhibition techniques are available, we found it difficult to acutely silence the orexinergic influence in the brain, presumably because orexin A is a stable peptide in the synaptic cleft and extracellular space. Therefore, the effects of orexin are presumably not attenuated after transient halorhodopsin-mediated inhibition of orexin neurons.

In this study, we applied a new pharmacogenetic technique, namely the DREADD technology [8], [9], [10], [14], to phamacogenetically manipulate the activity of orexin neurons. We found that pharmacogenetic stimulation of orexinergic tone resulted in an increased wakefulness time during the light period (rest phase). We also observed a modest effect even when we injected CNO in dark periods (Fig 3A, right panels). Chronic photo-stimulation (15 ms pulses at 20 Hz for 10 s every minute for 1 h) of orexin neurons has been reported to increase the number of NREM sleep-to-wake transitions but not to increase total wake time [6]. This suggests that pharmacogenetic stimulation as used here has an advantage in evoking more potent and chronic stimulation of orexin neurons than does ChR2-mediated photo-stimulation, although the time resolution of the former was not comparable to that of photostimulation.

The inhibition of orexin neurons by hM4D_i_ resulted in increased NREM sleep time during the dark period (active phase). DREADD utilizes G-protein coupled receptor signaling, which can affect neuronal activity in a relatively chronic and consistent manner, allowing investigation of the effects of orexin neuron inhibition. We confirmed this action by intracellular calcium imaging of neurons expressing DREADDs (Fig. S1). Compared with the effects of hM3D_q_ during the light periods, the effects of hM4D_i_ during the dark period were less striking. Since the orexin peptide is a highly stable substance, decreasing the activity of orexin neurons may not immediately induce a decrease in the stimulatory effects of orexin on target cells. We also observed modest effects of hM4D_i_ activation even when we administered CNO in the light period (Fig. 4A, left periods). This suggests that although orexinergic activity is lowered in the light period, further inhibition can affect sleep/wakefulness states.

Interestingly, we observed that DREADD-mediated inhibition resulted in shorter REM sleep time and longer REM latency one hour after CNO administration. These results could suggest an intriguing hypothesis that the activity of some population of orexin neurons might be necessary to evoke a proper REM sleep period in the rest period. This hypothesis should be further examined in future studies.

Our findings have profound meaning for sleep physiology, because many factors have been identified as influencing the activity of orexin neurons. For example, decreasing extracellular glucose concentration has been found to result in depolarization and increased discharge of orexin neurons, while increasing extracellular glucose concentration has been found to induce hyperpolarization and the cessation of discharge [21], [22]. Importantly, this mechanism is sufficiently sensitive to encode variations in glucose levels in the cerebrospinal fluid reflecting those occurring physiologically between normal meals [21], [22]. Therefore, changes in blood glucose concentration may influence an animal's vigilance states in daily life through orexin neurons.

Our findings have shown that specific changes in orexin neuronal activity can affect an animal's vigilance states. These results may enhance our understanding of the physiological relevance of orexin neurons in the regulation of sleep/wakefulness states.

## Materials and Methods

### Animals

All experimental procedures involving animals were approved by the Animal Experiment and Use Committee of Kanazawa University (AP-101567) and were in accordance with NIH 2009 guidelines. Generation of *Orexin-cre* mice was reported in our previous study [12]. Mice were maintained under a strict 12 hour light:dark cycle in a temperature- and humidity-controlled room and fed ad libitum. All efforts were made to minimize animal suffering and discomfort and to reduce the number of animals used.

### AAV production and purification

The plasmids *pcDNA5/FRT-HA-hM3Dq* and *pcDNA5/FRT-HA-hM4Di* [8] were each digested with PmeI, and the fragments containing *HA-hM3Dq* and *HA-hM4D* were ligated in the antisense direction into blunted AscI and NheI sites of *pAAV-double floxed-hChR2(H134R)-EYFP-WPRE-pA* [13], provided by Dr. Karl Deisseroth of Stanford University, to yield the plasmids *pAAV-DIO-HAhM3Dq* and *pAAV-DIO-HAhM4Di*, respectively.

Viruses were produced using a triple-transfection, helper-free method, and purified using a modification of a published protocol [23]. Briefly, 293A cells (Invitrogen), cultured in ten 100×20 mm cell culture dishes per viral vector, were transfected with pHelper (Stratagene), pACG-2-Y730F [24] (containing a mutant form of the *cap* gene of AAV2 and provided by Dr. Arun Srivastava of the University of Florida), and *pAAV-DIO-HAhM3Dq* or *pAAV-DIO-HAhM4Di*, using a standard calcium phosphate method. Three days later, the cells were collected, pelleted and resuspended in freezing buffer (10ml 0.15 M NaCl, 50 mM Tris, pH 8.0). After two freeze-thaw cycles and subsequent centrifugation, each lysate was treated with DNase I (40 µg/ml) and RNase A (40 µg/ml) and then with deoxycholic acid (Sigma) (0.5%), followed by filtration, as described previously [23]. Each cleared lysate was mixed with 1.5 ml of heparin-agarose suspension (Sigma) equilibrated with freezing buffer, incubated with gentle rotation for 60 min at 4°C, and loaded onto a Bio-Spin Column 100 (Bio-Rad Laboratories). Each column was washed three times with 2 ml freezing buffer, and viruses were eluted with 3 ml elution buffer (0.5 M NaCl, 50 mM Tris, pH 8.0). Each virus preparation was dialyzed against 1x PBS using Slide-A-Lyzer Dialysis Cassettes (Pierce) and concentrated using Concentration Solution (Pierce). The final purified viruses were stored at −80°C. The titers of rAAV-DIO-HAhM3Dq and rAAV-DIO-HAhM4Di were 5.7×10^12^ and 2.6×10^12^ genome copies/ml, respectively.

### Surgery

Male *orexin-cre* mice (10 to 12 weeks old) were anesthetized with sodium pentobarbital (0.5 mg/kg, i.p.) and positioned in a stereotaxic frame (David Kopf Instruments). Four holes were drilled into the skull of each mouse, at sites −1.4 mm posterior, ±0.9 mm lateral, and −5.5 mm ventral; and −1.8 mm posterior, ±0.9 mm lateral, and −5.7 mm ventral relative to the bregma (4 injection sites per mouse). A Hamilton needle syringe (33 gauge) was placed at each site, and 0.3 µl purified virus was delivered to each site over a 10-min period. After 5 min of rest, the needles were removed. The mice were sacrificed 14 days later, and tissue samples were assayed by immunohistochemistry. Following virus administration, an electrode for EEG and EMG recording was implanted to the skull of each mouse. The three arms of the electrode for EEG recording were placed approximately 2 mm anterior and 2 mm to the right, 2 mm posterior and 2 mm to the right, and 2 mm posterior and 2 mm to the left of the bregma. Stainless steel wires for EMG recording were sutured to the neck muscles of each mouse bilaterally, and each electrode was glued solidly to the skull.

Silicon tubes were implanted for remote CNO injection. The tip of a 30 cm-long silicon tube was inserted 1 cm into the peritoneal cavity and sutured to the abdominal wall. The other end of the silicon tube was placed outside the body through an incision in neck, and all incisions were sutured.

All animals were then housed individually for a recovery period of at least 7 days.

### Drugs and administration

Clozapine N-oxide (CNO; C0832, Sigma-Aldrich) was dissolved in saline to a concentration of 0.5 mg/ml. CNO was administered by intraperitoneal injection to each mouse (0.3 ml/30 g body weight) through the silicon tube, at ZT 4 in experiment 1 and at ZT 12 in experiment 2.

### Immunohistochemistry

Mouse brains were fixed and prepared as described previously [25]. For double immunofluorescence analysis, cryostat sections (40-µm thick) were preincubated for 1 h in 0.1 M phosphate buffer containing 1% bovine serum albumin and 0.25% Triton-X-100, and incubated overnight at 4°C with rabbit anti-orexin (1∶1000) and mouse anti-HA (Covance, 1∶1000) antibodies in the same solution. After three washes in the same solution, the sections were incubated with Alexa 594-conjugated donkey anti-rabbit IgG (Molecular Probes, 1∶500) and Alexa 488-conjugated goat anti-mouse IgG (Molecular Probes, 1∶800) for 90 min at room temperature. After three washes in 0.1 M phosphate buffer, the sections were mounted on glass slides and cover-slipped. Slides were examined by laser-confocal microscopy (Nikon, D-ECLIPSE C1si). For other immunohistochemical assays, cryostat sections (40-µm thick) were stained using the avidin-biotin-peroxidase method. Sections were washed for 1 h in 0.1 M phosphate buffer containing 1% bovine serum albumin and 0.25% Triton-X-100, incubated overnight at 4°C with mouse anti-HA antibody, washed three times, incubated for 1 h with biotinylated anti-mouse antibody (Vector Labs, Burlingame, CA, 1∶500), washed again and processed using a Vectastain ABC kit (Vector Labs, Burlingame, CA). After washing, the sections were mounted on glass slides and cover-slipped. To detect Fos immunoreactivity in orexin-expressing neurons, the sections were incubated with guinea pig anti-orexin antibody (Molecular Probes, 1∶1000; brown color) and rabbit anti-cFos antibody Ab-5 (Calbiochem, 1∶20000; black color). The numbers of cFos-positive and negative orexin-containing neurons were counted in coronal sections throughout the hypothalamic region by a single examiner who was blinded to treatment conditions, using an Olympus AX70 microscope. Cells were counted on both sides of the brain in consecutive 40-µm sections. Orexin neuron activity was scored as the percentage of double-labeled cells per animal.

### Sleep recordings

After the recovery period, animals were moved to a recording cage placed in an electrically shielded and sound-attenuated room. A cable for signal output was connected to the implanted electrode and animals were allowed to move freely. Signals were amplified through an amplifier (AB-611J, Nihon Koden, Tokyo) and digitally recorded on a PC using EEG/EMG recording software (Vital recorder, Kissei Comtec). Animals were allowed at least seven days to adapt to the recording conditions prior to any EEG/EMG recording session. Following the adaptation period, each animal was injected with both CNO and saline on separate experimental days with a 3-day interval. The order of injection (i.e. either CNO first or saline first) was randomized. EEG/EMG data were evaluated and staged for 24 hours on the day of CNO or saline administration. Data acquired on the day of saline administration were used as controls. After recording, the mice were again administered CNO and their brain sections were stained for Fos.

### Statistical Analysis

For statistical analyses of sleep/wake data, Student's paired t-tests were run under the same software described above. Data were expressed as mean±SEM. Differences were considered significant at P<0.05.

## Supporting Information

Figure S1 Typical example of responses of orexin neurons to application of CNO as measured by intracellular calcium imaging. *Orexin-YC2.1;orexin-Cre* double transgenic mice were injected with rAAV-DIO-HAhM3Dq (A) or rAAV-DIO-HAhM4Di (B). Two weeks later, brain slices from these mice were prepared and subjected to calcium imaging as described previously [11]. CNO application increased or decreased intracellular calcium levels of orexin neurons, respectively. Cholecystokinin-8S (CCK-8S) (A) or 5-HT (B) was used as positive controls.
(TIF)

Figure S2 Experimental procedures.
(TIF)

Figure S3 A representative one-hour hypnograms showing the effect of activation of orexin neurons showing effect of activation of orexin neurons by DREADD. A, Representative one-hour hypnograms for (A) wild type (WT) and (B) *orexin-cre* transgenic (TG) mice injected with rAAV-DIO-HAhM3Dq after CNO administration at ZT4.
(TIF)

Figure S4 A representative one-hour hypnograms showing the effect of activation of orexin neurons showing effect of inhibition of orexin neurons by DREADD. A, Representative one-hour hypnograms for (A) wild type (WT) and (B) *orexin-cre* transgenic (TG) mice injected with rAAV-DIO-HAhM4Di after CNO administration at ZT12.
(TIF)

## References

[1] Hagan JJ, Leslie RA, Patel S, Evans ML, Wattam TA (1999). Orexin A activates locus coeruleus cell firing and increases arousal in the rat.. Proc Natl Acad Sci U S A.

[2] Sakurai T (2007). The neural circuit of orexin (hypocretin): maintaining sleep and wakefulness.. Nat Rev Neurosci.

[3] Lee MG, Hassani OK, Jones BE (2005). Discharge of identified orexin/hypocretin neurons across the sleep-waking cycle.. J Neurosci.

[4] Takahashi K, Lin JS, Sakai K (2008). Neuronal activity of orexin and non-orexin waking-active neurons during wake-sleep states in the mouse.. Neuroscience.

[5] Mileykovskiy BY, Kiyashchenko LI, Siegel JM (2005). Behavioral correlates of activity in identified hypocretin/orexin neurons.. Neuron.

[6] Carter ME, Adamantidis A, Ohtsu H, Deisseroth K, de Lecea L (2009). Sleep homeostasis modulates hypocretin-mediated sleep-to-wake transitions.. J Neurosci.

[7] Adamantidis AR, Zhang F, Aravanis AM, Deisseroth K, de Lecea L (2007). Neural substrates of awakening probed with optogenetic control of hypocretin neurons.. Nature.

[8] Armbruster BN, Li X, Pausch MH, Herlitze S, Roth BL (2007). Evolving the lock to fit the key to create a family of G protein-coupled receptors potently activated by an inert ligand.. Proc Natl Acad Sci U S A.

[9] Alexander GM, Rogan SC, Abbas AI, Armbruster BN, Pei Y (2009). Remote control of neuronal activity in transgenic mice expressing evolved G protein-coupled receptors.. Neuron.

[10] Ferguson SM, Eskenazi D, Ishikawa M, Wanat MJ, Phillips PE (2011). Transient neuronal inhibition reveals opposing roles of indirect and direct pathways in sensitization.. Nat Neurosci.

[11] Tsujino N, Yamanaka A, Ichiki K, Muraki Y, Kilduff TS (2005). Cholecystokinin activates orexin/hypocretin neurons through the cholecystokinin A receptor.. J Neurosci.

[12] Matsuki T, Nomiyama M, Takahira H, Hirashima N, Kunita S (2009). Selective loss of GABA(B) receptors in orexin-producing neurons results in disrupted sleep/wakefulness architecture.. Proc Natl Acad Sci U S A.

[13] Tsai HC, Zhang F, Adamantidis A, Stuber GD, Bonci A (2009). Phasic firing in dopaminergic neurons is sufficient for behavioral conditioning.. Science.

[14] Krashes MJ, Koda S, Ye C, Rogan SC, Adams AC (2011). Rapid, reversible activation of AgRP neurons drives feeding behavior in mice..

[15] Estabrooke IV, McCarthy MT, Ko E, Chou TC, Chemelli RM (2001). Fos expression in orexin neurons varies with behavioral state.. J Neurosci.

[16] Yoshida Y, Fujiki N, Nakajima T, Ripley B, Matsumura H (2001). Fluctuation of extracellular hypocretin-1 (orexin A) levels in the rat in relation to the light-dark cycle and sleep-wake activities.. Eur J Neurosci.

[17] Vanni-Mercier G, Sakai K, Jouvet M (1984). Neurons specifiques de l'eveil dans l'hypothalamus posterieur du chat.. CR Acad Sci.

[18] Lu J, Bjorkum AA, Xu M, Gaus SE, Shiromani PJ (2002). Selective activation of the extended ventrolateral preoptic nucleus during rapid eye movement sleep.. J Neurosci.

[19] Sakurai T (2006). Roles of orexins and orexin receptors in central regulation of feeding behavior and energy homeostasis.. CNS Neurol Disord Drug Targets.

[20] Yoshida K, McCormack S, Espana RA, Crocker A, Scammell TE (2006). Afferents to the orexin neurons of the rat brain.. J Comp Neurol.

[21] Yamanaka A, Beuckmann CT, Willie JT, Hara J, Tsujino N (2003). Hypothalamic orexin neurons regulate arousal according to energy balance in mice.. Neuron.

[22] Burdakov D, Gerasimenko O, Verkhratsky A (2005). Physiological changes in glucose differentially modulate the excitability of hypothalamic melanin-concentrating hormone and orexin neurons in situ.. J Neurosci.

[23] Auricchio A, Hildinger M, O'Connor E, Gao GP, Wilson JM (2001). Isolation of highly infectious and pure adeno-associated virus type 2 vectors with a single-step gravity-flow column.. Hum Gene Ther.

[24] Zhong L, Li B, Mah CS, Govindasamy L, Agbandje-McKenna M (2008). Next generation of adeno-associated virus 2 vectors: point mutations in tyrosines lead to high-efficiency transduction at lower doses.. Proc Natl Acad Sci U S A.

[25] Nambu T, Sakurai T, Mizukami K, Hosoya Y, Yanagisawa M (1999). Distribution of orexin neurons in the adult rat brain.. Brain Res.

