# Supplementary figures and images for: Pharmacogenetic Modulation of Orexin Neurons Alters Sleep/Wakefulness States in Mice
Source: PLoS One. 2011 May 27;6(5):e20360. doi: 10.1371/journal.pone.0020360 (PMC3103553; doi:10.1371/journal.pone.0020360)

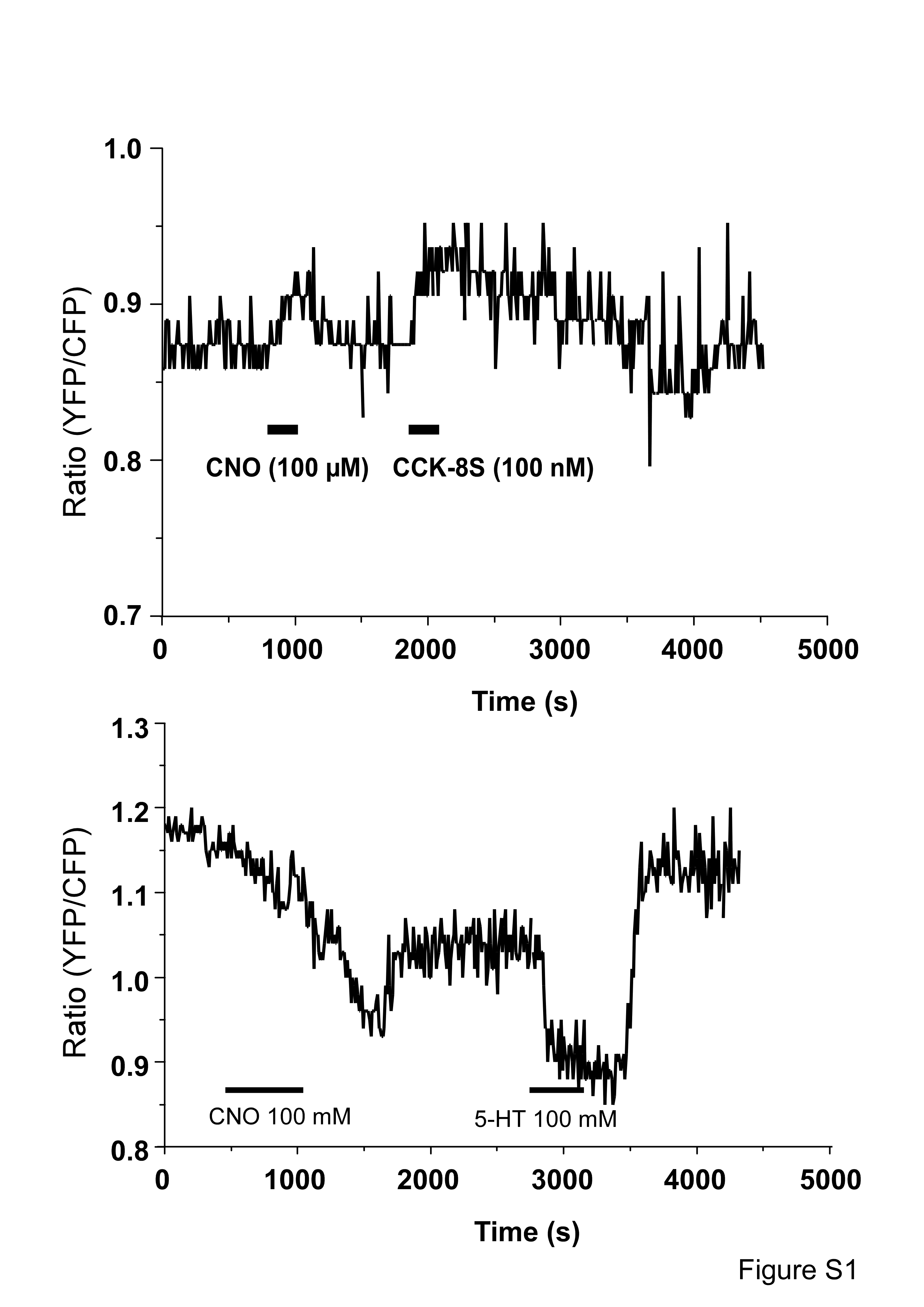

Supplement: Figure S1 — Typical example of responses of orexin neurons to application of CNO as measured by intracellular calcium imaging. Orexin-YC2.1;orexin-Cre double transgenic mice were injected with rAAV-DIO-HAhM3Dq (A) or rAAV-DIO-HAhM4Di (B). Two weeks later, brain slices from these mice were prepared and subjected to calcium imaging as described previously [11]. CNO application increased or decreased intracellular calcium levels of orexin neurons, respectively. Cholecystokinin-8S (CCK-8S) (A) or 5-HT (B) was used as positive controls. (TIF) [file pone.0020360.s001.tif]

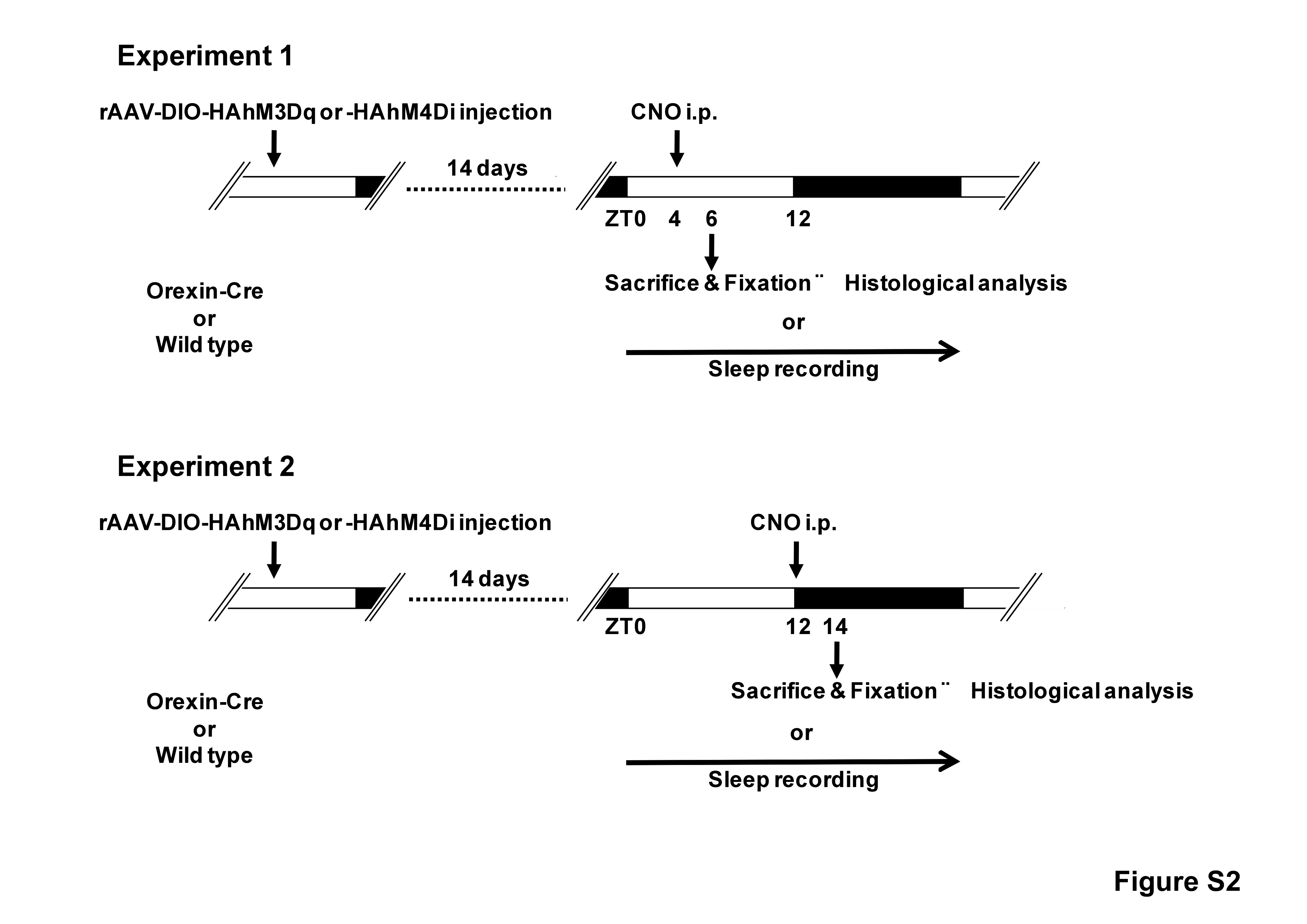

Supplement: Figure S2 — Experimental procedures. (TIF) [file pone.0020360.s002.tif]

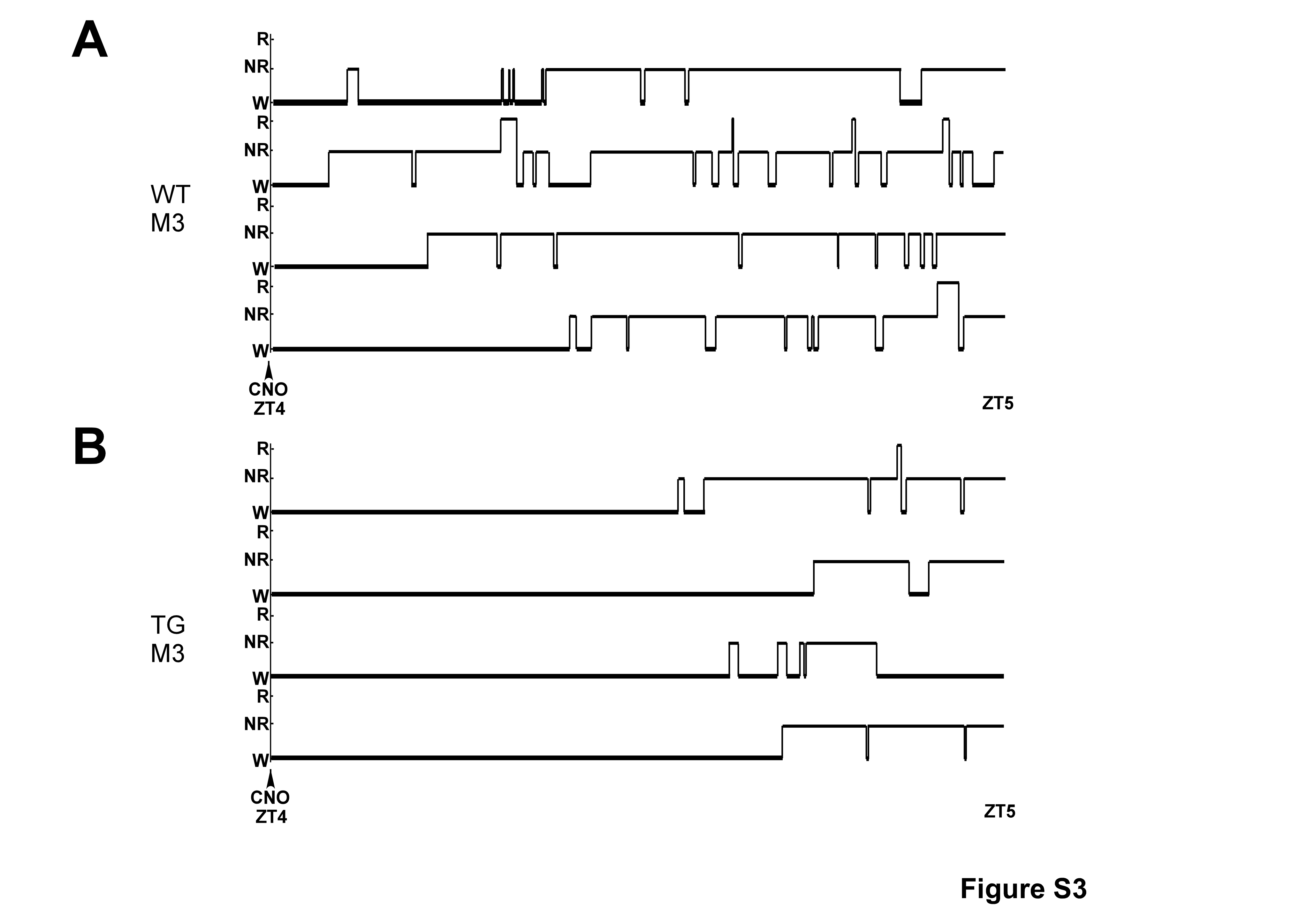

Supplement: Figure S3 — A representative one-hour hypnograms showing the effect of activation of orexin neurons showing effect of activation of orexin neurons by DREADD. A, Representative one-hour hypnograms for (A) wild type (WT) and (B) orexin-cre transgenic (TG) mice injected with rAAV-DIO-HAhM3Dq after CNO administration at ZT4. (TIF) [file pone.0020360.s003.tif]

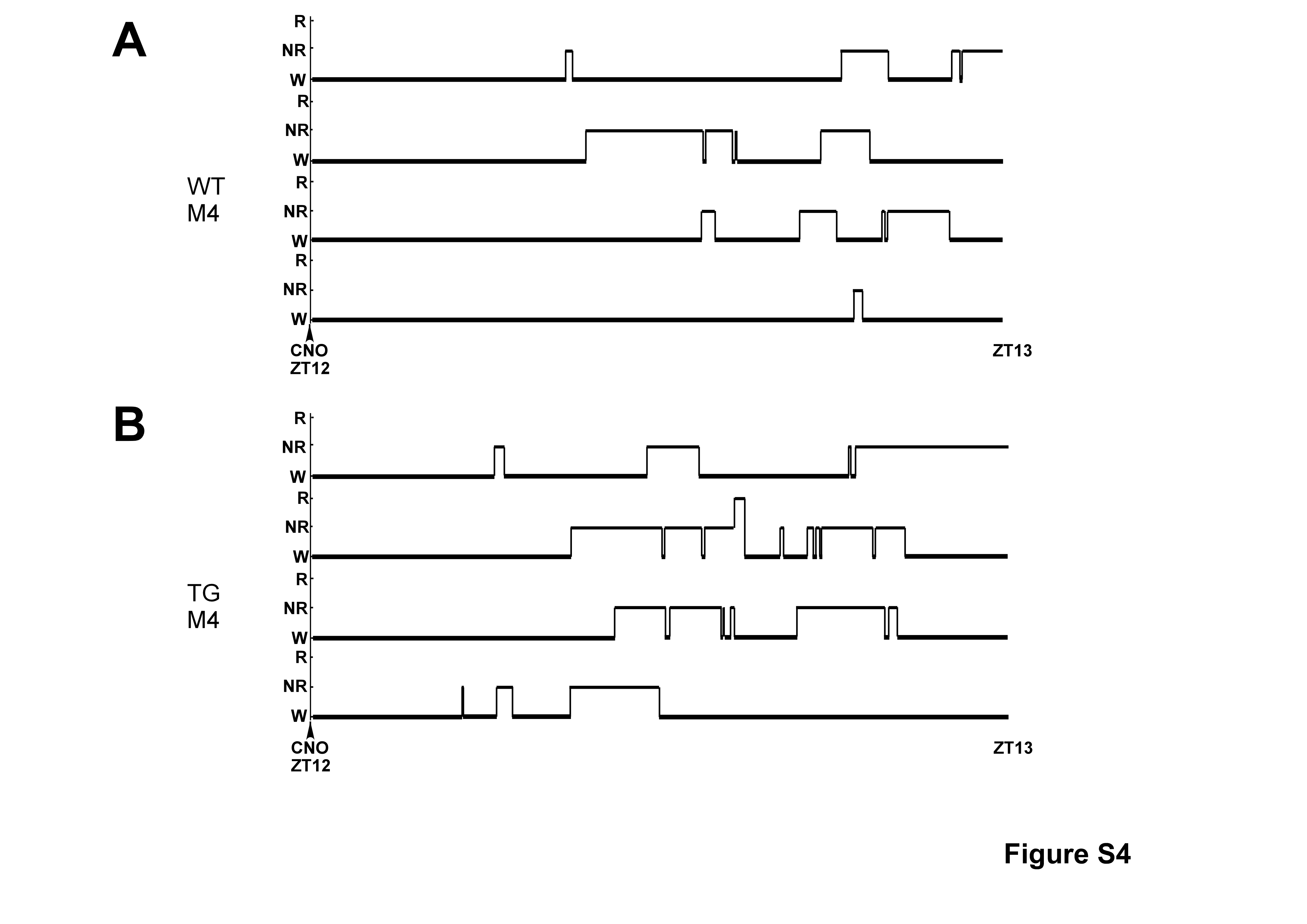

Supplement: Figure S4 — A representative one-hour hypnograms showing the effect of activation of orexin neurons showing effect of inhibition of orexin neurons by DREADD. A, Representative one-hour hypnograms for (A) wild type (WT) and (B) orexin-cre transgenic (TG) mice injected with rAAV-DIO-HAhM4Di after CNO administration at ZT12. (TIF) [file pone.0020360.s004.tif]
